# Lymphocyte-Activation Gene 3 (LAG3) Protein as a Possible Therapeutic Target for Parkinson’s Disease: Molecular Mechanisms Connecting Neuroinflammation to α-Synuclein Spreading Pathology

**DOI:** 10.3390/biology9040086

**Published:** 2020-04-23

**Authors:** Efthalia Angelopoulou, Yam Nath Paudel, Chiara Villa, Mohd. Farooq Shaikh, Christina Piperi

**Affiliations:** 1 Department of Biological Chemistry, Medical School, National and Kapodistrian University of Athens, 11527 Athens, Greece; angelthal@med.uoa.gr; 2 Neuropharmacology Research Strength, Jeffrey Cheah School of Medicine and Health Sciences, Monash University Malaysia, Bandar Sunway 47500, Selangor, Malaysia; yam.paudel@monash.edu (Y.N.P.); Farooq.shaikh@monash.edu (M.F.S.); 3 School of Medicine and Surgery, University of Milano-Bicocca, 20900 Monza, Italy; chiara.villa@unimib.it

**Keywords:** LAG3, Parkinson’s disease, α-synuclein, neuroinflammation, biomarker

## Abstract

Parkinson’s disease (PD) is the most common neurodegenerative movement disorder without any objective biomarker available to date. Increasing evidence highlights the critical role of neuroinflammation, including T cell responses, and spreading of aggregated α-synuclein in PD progression. Lymphocyte-activation gene 3 (LAG3) belongs to the immunoglobulin (Ig) superfamily expressed by peripheral immune cells, microglia and neurons and plays a key role in T cell regulation. The role of LAG3 has been extensively investigated in several human cancers, whereas until recently, the role of LAG3 in the central nervous system (CNS) has been largely unknown. Accumulating evidence highlights the potential role of LAG3 in PD pathogenesis, mainly by binding to α-synuclein fibrils and affecting its endocytosis and intercellular transmission, which sheds more light on the connection between immune dysregulation and α-synuclein spreading pathology. Serum and cerebrospinal fluid (CSF) soluble LAG3 (sLAG3) levels have been demonstrated to be potentially associated with PD development and clinical phenotype, suggesting that sLAG3 could represent an emerging PD biomarker. Specific single nucleotide polymorphisms (SNPs) of the LAG3 gene have been also related to PD occurrence especially in the female population, enlightening the pathophysiological background of gender-related PD clinical differences. Given also the ongoing clinical trials investigating various LAG3-targeting strategies in human diseases, new opportunities are being developed for PD treatment research. In this review, we discuss recent preclinical and clinical evidence on the role of LAG3 in PD pathogenesis and biomarker potential, aiming to elucidate its underlying molecular mechanisms.

## 1. Introduction

Parkinson’s disease (PD) is the most common neurodegenerative movement disorder affecting approximately 1–2% of the population above the age of 65 years [1]. The key pathological hallmarks of the disease involve the intracellular accumulation of α-synuclein in the form of Lewy bodies and Lewy dendrites, as well as the dopaminergic neuronal loss in the substantia pars compacta (SNpc) resulting in nigrostriatal degeneration [1,2,3]. However, its pathology progresses gradually from the brainstem to cortical regions [4].

Although many genetic and environmental factors have been associated with PD development, the exact etiology of the disease remains obscure. Along with autophagy impairment, mitochondrial dysregulation and oxidative damage, accumulating evidence suggests that neuroinflammation, including T cell-driven immune responses, as well as neuron-to-neuron transmission of aggregated α-synuclein may significantly contribute to PD progression [5,6]. Given the absence of a reliable, objective and easily measured biomarker for PD, a growing number of studies have been focusing on the investigation of novel diagnostic and prognostic biomarker candidates, such as α-synuclein, dopamine metabolites, neuropeptide hormones like orexin, apolipoprotein A1, DJ-1, various molecules associated with proteasomes and micro-RNAs, as well as proinflammatory cytokines [1,7]. However, the results of many of these studies are inconsistent, and there is still no available serum or cerebrospinal fluid (CSF) molecule that could be effectively used for PD diagnosis or progression [1].

**Figure 1:**
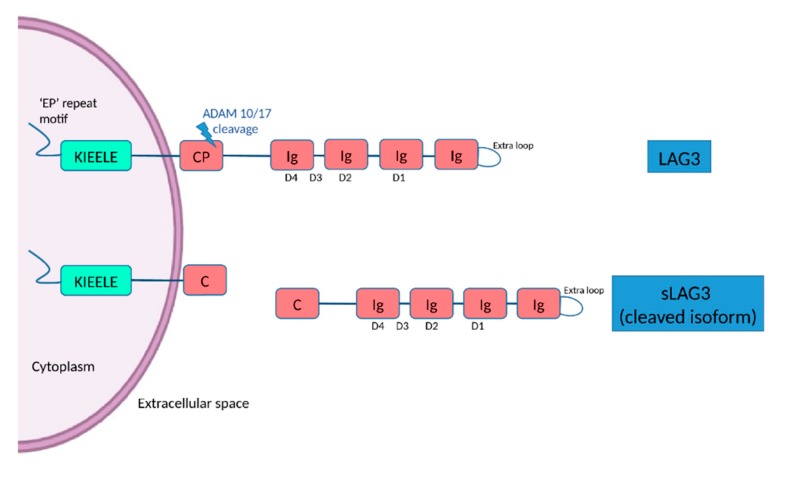
The structure of LAG3 and its cleaved soluble form (sLAG3). LAG3 is a transmembrane protein consisting of 498 amino acids, with four extracellular Ig-like domains (D1–D4) and an intracellular domain, containing an ‘EP’ (glutamic acid-proline) repeat responsible for anchorage and a KIEELE motif, which is required for the LAG3-mediated negative inhibitory signal. LAG-3 can be cleaved within the transmembrane domain at the connecting peptide (CP) by two members of metalloproteases known as ADAM 10 and ADAM 17 to release sLAG-3, which contributes to the regulatory function of LAG-3.

Lymphocyte-activation gene 3 (*LAG3*, also termed as CD223) is located to chromosome 12p13 and belongs to the immunoglobulin (Ig) superfamily expressed by peripheral immune cells including subsets of natural killer cells, CD4^+^ and CD8^+^ T cells, B cells and dendritic cells, as well as microglial cells and neurons [8,9]. LAG3, firstly identified in 1990 [10], is a transmembrane protein consisting of 498 amino acids, with four extracellular Ig-like domains (D1–D4) and an intracytoplasmic part [11]. The D1 domain contains a unique extra loop and a small amino acid sequence that confers binding to major histocompatibility complex (MHC) class II molecules (MHC II). The KIEELE motif, a unique amino acid sequence in the intracellular domain is required for the LAG3-mediated negative inhibitory signal on T cells whereas and ‘EP’ repeat motif mediates anchorage (Figure 1) [11]. The transmembrane connecting peptide (CP) of LAG3 can be cleaved by the metalloproteinases ADAM10 and ADAM17 to produce its soluble form, detectable in serum. LAG3 is a CD4 homologue, and binds to MHC class II molecules on antigen presenting cells with higher affinity than CD4 [11,12]. Being an immune checkpoint receptor, LAG3 regulated T cell immune responses and immune homeostasis, mainly by inhibiting T cell activation and proliferation [9]. LAG3 is highly expressed in peripheral immune organs including the spleen and the thymus, and also in the central nervous system (CNS) [13]. LAG3 could be expressed on neuronal cells [9], but also on microglia [14]. Although there is evidence on the role of LAG3 in other disorders, especially autoimmune diseases and cancer [11], the function of LAG3 in the CNS is largely unknown [15], and only recently, some experimental studies have begun to shed light on its role in neurodegenerative diseases.

Given the implication of LAG3 in immune regulation, it may play an important role in the neuroinflammatory processes underlying the pathogenesis of PD, by affecting the cell-to-cell transmission of α-synuclein in a “prion-like” manner [16]. It has been also revealed that soluble LAG3 (sLAG3) can be used as a PD biomarker candidate.

In this review, we discuss recent preclinical and clinical evidence on the role of LAG3 in PD pathogenesis and biomarker potential, aiming to elucidate its underlying molecular mechanisms.

## 2. The Role of α-Synuclein Spreading and Neuroinflammation in PD Pathogenesis: Connecting the Dots

A growing body of preclinical and clinical evidence suggests that spreading of α-synuclein in a “prion-like” manner may be highly involved in PD progression [17]. In particular, aggregated α-synuclein appears to spread in a seemingly stereotypical topographical manner in PD patients, often referred to as Braak staging [4,18]. According to this hypothesis, Lewy bodies were initially present in the gut or the olfactory bulb, via which they gradually spread to the brainstem and eventually, to the cortex [19]. Lewy bodies with α-synuclein were found within grafted neurons from PD patients subjected to transplantation of fetal mesencephalic dopaminergic cells, suggesting a potential host-to-graft propagation of the disease [20]. Another in vivo study has indicated that α-synuclein could be transferred from the brain of a rat model overexpressing human α-synuclein into grafted dopaminergic neurons via endocytic pathways [21]. In this context, the inoculation of nigral Lewy body extracts obtained from postmortem brains of PD patients into the SN or striatum of mice and monkeys resulted in nigrostriatal degeneration, internalization of the exogenous α-synuclein within the host neuronal cells and in pathological aggregation of the endogenous α-synuclein in the recipient animals [22]. The existence of a similar widespread mechanism is further described for other proteins prone to aggregate in other neurodegenerative diseases, such as beta-amyloid in the pathogenesis of Alzheimer’s disease (AD) [23]. The exact mechanisms underlying the exit of α-synuclein from the host cell and the transmission to the recipient cell are still unclear. Emerging evidence indicated that α-synuclein is detected in extracellular biological fluids and is rapidly secreted from cells. Moreover, the extracellular α-synuclein is internalized by lipid raft-mediated endocytosis, interacting with the specific monoganglioside GM1, a component of these membrane domains [24,25]. Using α-synuclein-enriched models, it has been also demonstrated that mesenchymal stem cells could suppress the cell-to-cell transmission of extracellular α-synuclein by inhibiting its clathrin-mediated endocytosis, leading to a protective effect on cortical and dopaminergic neurons [26].

Growing evidence over the past decades has shown that both innate and adaptive immune dysfunction, mainly involving microglia and T cell responses respectively, may play a key role in PD pathogenesis. In particular, larger amounts of microglia have been reported in the SNpc of PD patients compared to controls [27]. Higher proportion of non-classical monocytes, activated T cells, as well as increased levels of the proinflammatory cytokines TNF-α, IL-2 and IL-6 have been identified in the CSF of PD patients compared to controls [28]. In accordance, CD4^+^ and CD8^+^ T cells infiltrated the brain, as observed in postmortem human PD tissue samples as well as in the 1-methyl-4-phenyl-1,2,3,6-tetrahydropyridine (MPTP) mouse PD models [29]. Moreover, α-synuclein-derived peptides induced T cell-mediated responses in animal models [30], and MHC II knockout mice overexpressing α-synuclein have been protected against dopaminergic neuronal loss [31]. Another study has shown that extracellular α-synuclein-derived fragments may be acquired by brain cells during disease pathogenesis and act as antigenic epitopes presented to T cells, triggering T helper and cytotoxic immune responses in patients with PD [32]. This process may explain the association of PD with alleles of the acquired immune system. Hence, α-synuclein transmission and immune dysregulation may jointly contribute to PD pathogenesis, and there is a need for greater understanding of the potential connecting molecular pathways involved.

## 3. The Implication of LAG3 in PD Pathogenesis

Increasing evidence highlights the fact that proteins traditionally considered to act in the peripheral immune system may actually play critical roles in the CNS and subsequently be implicated in neurological disorders [15]. Being a mediator of T cell regulation and immune response, LAG3 has been recently suggested to be involved in PD pathogenesis and specifically in α-synuclein transmission.

Notably, a study that used preformed fibrils (PFFs) of recombinant mouse α-synuclein as a model to investigate the neuron-to-neuron transmission of α-synuclein, has demonstrated that LAG3 may play an important role in this process [15]. Among the three identified transmembrane protein candidates for binding to exogenous α-synuclein PPFs after unbiased screening, LAG3 exhibited the highest selectivity for α-synuclein PPFs compared to α-synuclein monomers [15]. Further analysis revealed that out of the four Ig-like domains of LAG3, D1 was responsible for binding to α-synuclein PPFs, particularly via its residues 52–109 [15]. LAG3 also triggered α-synuclein PFFs endocytosis, since LAG3 deletion could significantly reduce the internalization of α-synuclein PFFs in mouse cortical neurons [15]. The early endosomal marker RAB5, a small Ras-like GTPase, colocalized with α-synuclein PFFs in this experiment [15], and there has been already reported that Rab5 is fundamentally implicated in α-synuclein endocytosis [33]. Besides that, LAG3 overexpression enhanced the phosphorylation of α-synuclein at serine 129 [15], a process that has been already associated with PD pathology [34]. Furthermore, α-synuclein PPFs-induced synaptic disruption was inhibited in LAG3 knockout in vitro, since the reduction of SNAP25 and synapsin II was prevented compared to wild-type cells [15]. LAG3 deletion inhibited α-synuclein neuronal transmission, α-synuclein PPFs-induced neuronal loss and toxicity in vitro, accompanied by lower intracellular calcium levels [15]. In particular, signaling via the intracellular domain of LAG3 could possibly mediate α-synuclein-induced neurotoxicity, since the deletion of this domain was found to reduce α-synuclein PPFs-induced toxic effects [15]. LAG3 deletion or the usage of LAG3 antibodies also decreased the α-synuclein-PPFs-induced neurotoxicity in human A53T α-synuclein transgenic neuronal cell cultures, further highlighting the implication of LAG3 in the progression of human α-synuclein pathology [15]. Additionally, LAG3 was expressed only in neurons and not in microglia or astrocytes in this study, suggesting that the abovementioned proposed mechanism for α-synuclein transmission may be neuron-specific [15].

Apart from in vitro evidence, LAG3 has been significantly involved in α-synuclein transmission and related neurotoxicity in vivo [15]. Phosphorylated α-synuclein staining was significantly reduced in the dopaminergic neurons of SNpc after injection of α-synuclein PFFs into the striatum of LAG3 knockout mice, compared to wild-type ones [15]. The α-synuclein PPFs-induced dopaminergic neuronal loss, along with the reduction of dopamine and its metabolites (3MT, DOPAC and HVA), as well as the decrease of TH and DAT enzymes were delayed in the SNpc of the LAG3 knockout animals [15]. Of note, the behavioral impairment of the animals was also prevented in the case of LAG3 deletion, as evaluated by grip strength and the pole test [15] that is considered to effectively reflect dopaminergic function [35]. Collectively, these interesting findings suggest that LAG3 could bind to extracellular α-synuclein fibrils, be involved in the initiation of α-synuclein endocytosis and transmission, as well as contribute to α-synuclein-induced dopaminergic neuronal loss and neurotoxicity. Therefore, LAG3 protein may play a fundamental role in α-synuclein spreading pathology and neurodegeneration in PD, and further studies are needed to investigate the exact underlying molecular mechanisms.

Parkinson’s disease shares some common features with prion diseases, including abnormal protein aggregation, neuronal loss and microglia activation. However, a recent in vivo study has shown that although LAG3 levels were increased after prion infection in mice, LAG3 knockout did not affect PrPSc load, microglia activation, astrocyte reaction, expression of inflammatory genes and degree of neurodegeneration in the animal models, suggesting that LAG3 may not be implicated in the pathogenesis of prion diseases [9]. Nevertheless, α-synuclein and PrPSc differ significantly in terms of molecular structure and function, and the underlying pathophysiology of PD and prion diseases, including Creutzfeldt–Jakob disease (CJD) is not identical [36]. The lack of LAG3 involvement in the pathogenesis of prion disease may further support the fact that it is specific for PD, and potentially able to differentiate from “pure” prionopathies, such as CJD.

## 4. LAG3 as a Potential Biomarker for PD

The sLAG3 is produced either by alternative splicing of LAG3 RNA or cleaved and released from the full-length LAG3 protein [12,37]. A recent study in the Chinese population has indicated that serum sLAG3 levels measured by quantitative enzyme linked immunosorbent assay (ELISA) were significantly higher in PD patients compared to age- and gender-matched controls and to patients with essential tremor, another common movement disorder [7]. Its estimated sensitivity and specificity was about 82% and 78% versus controls, and 76% and 82% versus patients with essential tremor, respectively [7]. Increased serum sLAG3 levels were also found to be associated with more severe non-motor symptoms as evaluated by non-motor symptoms scale (NMSS) scores, as well as excessive daytime sleepiness [7]. In this regard, increased CSF levels of YKL-40, an inflammatory marker, have been correlated with faster cognitive impairment in PD [38], and the concentration of tumor necrosis factor-α, interleukin-1β and nitric oxide in the CSF have been associated with probable REM sleep behavior disorder in PD patients [39]. These findings suggest that excessive inflammation may accompany non-motor symptoms of PD, and LAG3, as an indicator of cellular immune responses, may reflect the increased inflammation related to non-motor PD manifestations [7]. Taken together, apart from its value as a diagnostic biomarker, serum sLAG3 may be also associated with PD clinical phenotypes.

Based on these interesting evidences, another larger study aimed to additionally explore the potential role of sLAG3 in the CSF as a potential PD biomarker in a Chinese cohort [40]. More specifically, it was demonstrated that the mean sLAG3 levels in CSF were lower in PD cases compared to healthy controls, whereas no significant differences were observed in the case of serum sLAG3 levels between these groups [40]. These results are in disagreement with the findings of the previously mentioned study, in which serum sLAG3 levels were increased in PD patients compared to controls. This discrepancy could be at least partially explained by the different methods used for the evaluation of sLAG3 levels in each study (ELISA versus mesoscale discovery electrochemiluminescence immunoassay (MSD-ECL). Indeed, the MSD-ECL technology displays higher repeatability, stability, sensitivity and homogeneity than ELISA assay [41]. Gender, age, disease duration and severity as assessed by Hoehn and Yahr PD staging, did not significantly affect sLAG3 concentration in CSF of PD patients [40]. The estimated sensitivity and specificity of CSF sLAG3 as a possible PD biomarker were about 45% and 90%, respectively [40]. Although the sensitivity is low, this is the result of a single study applying MSD-ECL in CSF samples, where concentration of the antigen may be low or the affinity reagents may need better standardization. However, the high estimated specificity of CSF sLAG3 is very encouraging [40].

Although the results of studies investigating the role of α-synuclein as a potential biomarker are inconsistent, several lines of evidence have shown that CSF α-synuclein levels may be lower in PD patients compared to controls [42,43]. Given the possible implication of LAG3 in α-synuclein propagation, the relationship between sLAG3 and α-synuclein levels in the CSF has been also investigated [40]. In particular, although CSF sLAG3 levels were positively correlated with the CSF levels of α-synuclein in controls, no significant associations were found in PD patients [40]. It is important to mention that the two subtypes of LAG3 protein (full-length LAG3 and sLAG3) may exert distinct roles in immune regulation. More specifically, full-length membrane LAG3 generally suppresses T cell immune responses [44], whereas membrane LAG3 blockade or proteolytic cleavage, induces T cell activation and production of cytokines [45]. sLAG3 has been also demonstrated to inhibit the differentiation of monocytes to both macrophages and dendritic cells [46], whereas LAG3 blockade has been reported to act as an enhancer of T cell response in the case of antitumor cell vaccination [47]. Although the exact roles of LAG3 and sLAG3 in the CNS and PD are still unknown, based on the results of the study by Guo and colleagues, it has been speculated that LAG3 and sLAG3 imbalance in the brain may create an immunologically impaired microenvironment, susceptible to α-synuclein-mediated neurodegeneration, which the aggregated α-synuclein could utilize in order to spread in the CNS [40].

As discussed above, α-synuclein transmission may be at least partially responsible for the observed anatomical and temporal progression of PD pathology [48]. In this context, the potential relationship between the regional gene expression and brain atrophy was investigated by a study comparing genetic data with high quality regional imaging. The seventeen top candidate genes identified by genome wide association studies (GWAS) as well as genes related to autosomal recessive PD and implicated in trans-synaptic α-synuclein transfer, including *RAB5A*, *NRXN1*, *APLP1* and *LAG3*, were included in the analysis [49]. Regional PD atrophy patterns were derived from magnetic resonance imaging (MRI) obtained from the Parkinson's progression markers initiative (PPMI) whereas regional microarray expression evidence was collected from the Allen Brain Atlas, containing data from healthy controls. Among all tested gene candidates, the expression patterns of *LAG3* and *RAB5A*, which are involved in α-synuclein transmission, were the two most significant “predictors” of regional atrophy in PD. In particular, the level of expression of *LAG3* gene was positively correlated with regional atrophy, whereas the inverse relationship was shown for *RAB5A* gene, possibly suggesting an inhibitory role in PD neurodegeneration. Given the fact that the detected expression pattern of these genes is also present in the healthy normal brain, the authors suggested that these pre-existing regionally different expression profiles may be exploited for the spatial progression of PD pathology after the onset of the disease, and do not reflect initiating pathogenic mechanisms [49].

**Figure 2:**
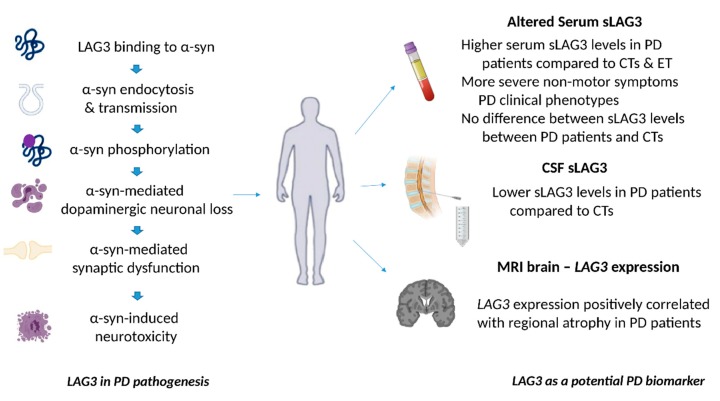
The role of LAG3 in the pathogenesis of Parkinson’s disease and its biomarker potential. LAG3 can bind to α-synuclein and trigger its endocytosis, contributing to α-synuclein cell-to-cell transmission. Overexpression of LAG3 may enhance the phosphorylation of α-synuclein at serine 129. LAG3 has been also implicated in α-synuclein dopaminergic neuronal loss, neurotoxicity and synaptic dysfunction. Clinical evidence has shown that soluble LAG3 (sLAG3) levels may be higher in patients with PD compared to controls (CTs) or patients with essential tremor (ET), whereas there is also evidence indicating that serum sLAG3 levels display no difference between PD patients and CTs. Lower sLAG3 levels in the cerebrospinal fluid (CSF) have been associated with PD development, and *LAG3* gene expression has been also correlated with regional atrophy in magnetic resonance imaging (MRI) brain scans of patients with PD.

The occipital lobes were reported to exhibit the greatest atrophy in this study, followed by the temporal lobes and basal ganglia [7]. Interestingly, visual hallucinations were found to be related with selective brain atrophy of the LAG3 high expressing cuneus and lingual gyrus of the occipital lobe in the examined PD population, and these brain regions have already been associated with PD-related hallucinations in previous reports [50]. The degree of cognitive decline in PD has been associated with the degree of temporal atrophy in other studies [51]. Collectively, these combined imaging-genetic findings suggest that the higher regional *LAG3* gene expression may be associated with regional atrophy in PD, and could also be correlated with the degree of non-motor manifestations, including psychotic symptoms, which are in agreement with the results of the abovementioned study investigating sLAG3 levels in the serum of PD patients [7]. Notably, as imaging and genetic data were obtained from different populations (PD patients and healthy controls respectively) and the possibility that gene expression patterns may significantly differ in the case of PD, further studies are obviously needed to validate the results of the abovementioned study (Figure 2).

## 5. Single Nucleotide Polymorphisms (SNPs) of the *LAG3* Gene and the Risk of PD Development

It has been already demonstrated that specific SNPs of the *LAG3* gene, including rs951818, rs1922452 and rs870849, were associated with increased susceptibility to multiple sclerosis [52,53]. Interestingly, mutations in gene encoding proteins being able to bind α-synuclein, such as the neuron-specific α3-subunit of Na^+^/K^+^-ATPase have been associated with rapid-onset dystonia Parkinsonism (RDP) [54]. In this regard, a very recent case-control study aiming to identify potential relationships between the abovementioned *LAG3* SNPs and PD in a Chinese population did not reveal any significant associations between these *LAG3* SNPs and PD development [40]. However, after stratification analysis, the frequency of rs951818-CC and rs1922452-AA alleles was higher in women with PD, compared to female healthy controls in this study [40]. Of note, these specific *LAG3* SNPs are located within the intron-spanning reads of the RNA-seq, which play a key role in splicing [40]. This fact suggests and further supports the hypothesis that improper alternative splicing of LAG3 and subsequent LAG3-sLAG3 imbalance may be involved in immune dysregulation, α-synuclein transmission and increased PD risk [40].

Male gender is one of the most significant risk factors for PD development, since men display a 2-fold higher risk for PD compared to women [55]. However, women with early menopause have been demonstrated to have an increased PD risk [56]. Besides that, gender-related differences have been observed in respect to the clinical phenotype of PD [57], as well as postmortem specific gene expression profiling of dopaminergic neurons from PD patients [58,59]. In particular, among others, TNF-receptor associated protein 1 (TRAP1) and the TNF receptor 1A protein being implicated in inflammatory responses were differently expressed between men and women with PD [59]. Preclinical evidence has also demonstrated that estrogens may play an important role in the regulation of microglial activity in the case of PD, since the MPTP-induced neurotoxicity of activated microglial cells was correlated with serum estrogen levels [55]. Based on such findings, these specific *LAG3* SNPs may increase PD risk in women by altering the estrogen-related microglial activation and immune responses [40].

## 6. Discussion and Perspectives

Collectively, increasing evidence highlights the potential role of LAG3 in PD pathogenesis, primarily by binding to α-synuclein and affecting its endocytosis and intercellular transmission. Clinical evidence has shown that serum and CSF sLAG3 may be associated with PD development and a clinical phenotype, suggesting that sLAG3 could represent an emerging PD biomarker. Specific SNPs of the *LAG3* gene have been also related to PD in the female population, shedding more light in the pathophysiological background of gender-related PD clinical differences. However, although there is ongoing research in this field, the implication of LAG3 in PD is still in its infancy, and the downstream pathways of LAG3-mediated α-synuclein transmission are still unexplored. Towards this direction, the researchers of Dawson’s lab are currently investigating the LAG3-related signaling pathways and interacting molecules [60].

It is important to mention that although LAG3 binds to α-synuclein and critically contributes to its transmission, other proteins binding α-synuclein fibrils with some specificity have recently been identified. Among them, some studies have identified neurexin-1β and amyloid beta precursor-like protein 1 (APLP1) as putative receptors for fibrillary α-synuclein, although with lower selectivity [15]. Furthermore, the endoplasmic reticulum-associated deubiquitylase USP19 acts as a chaperone and mediates the recruitment of misfolded α-synuclein proteins to Rab9-positive late endosomes prior to secretion [61]. In addition to LAG3, the α3-subnit of the Na^+^/K^+^-ATPase (α3NKA) is another cell surface protein expressed in neurons that binds to extracellular α-synuclein assemblies, with high affinity for α-synuclein fibrils. Although the role of α3-NKA in α-synuclein endocytosis was not investigated, it seems that clustering of α-synuclein at the membrane induced the redistribution of α3-NKA, thus reducing its ability to pump out Na^+^ from neurons [54]. Heparan sulfate proteoglycans which are abundant extracellular glycoproteins, bind to α-synuclein fibrils and promote their uptake [62], while the high mobility group protein 1 (HMGB1) binds to aggregated α-synuclein [63,64]. In addition, α-synuclein fibrils may also be released and transmitted from neuron-to-neuron via exosomes and membranous bridges generated by tunneling nanotubes [65]. Besides that, α-synuclein oligomers can penetrate cell membrane via pore-like formations, inducing local structural rearrangements of the proteins and therefore disrupting the lipid packaging [66]. The cellular prion protein (PrPC) binds to beta-amyloid oligomers, contributing to neurotoxicity, [67] and has been also recently involved in α-synuclein-mediated pathology. Although the presence of PrPC was not necessary for the pathological α-synuclein spreading, the expansion of α-synuclein was faster in the case of PrPC-overexpressing mouse models, suggesting its potential role in the modulation of α-synuclein fibrils [68,69]. Given these findings, as well as the fact that the lack of LAG3 does not completely disrupt α-synuclein transmission [15], it is reasonable to speculate that LAG3 is not the only mediator of α-synuclein endocytosis and transmission, and its interaction with the other potential candidates mentioned above should be considered and further investigated.

Furthermore, genome wide association studies have revealed that specific MHC II gene haplotypes, such as DRB1*15:01 and DRB5*01 alleles, as well as polymorphisms of non-coding genetic regions that could increase MHC II expression levels may be associated with PD development [70]. In addition, microglia expressing MHC II have been identified in the SNpc of patients with PD, and their detected levels were associated with dopaminergic degeneration [71]. Apart from MHC II molecules, also galectin-3 represents a LAG3 ligand. Serum galectin-3 levels were detected significantly higher in PD patients compared to controls, and positively correlated with Hoehn and Yahr stages of PD [72]. Galectin-3 was also highly implicated in α-synuclein-induced activation of primary microglial cells from mice [73]. Hence, these additional findings further support the potential role of LAG3 in PD, and note the need for further investigation of the relationship between LAG3 and MHC II molecules, galectin-3 and liver sinusoidal endothelial cell lectin (LSECtin, another LAG3 identified ligand) [11], especially in PD pathogenesis.

It has been demonstrated that LAG3 proteolytic cleavage can be carried out by ADAM17 and ADAM10, two membrane metalloproteases, resulting in the generation of sLAG3 [74]. Interestingly, extracellular α-synuclein may downregulate the expression of *ADAM10*, whose role has been already established in Alzheimer’s disease pathogenesis [75]. Thus, a low activity of ADAM17 and ADAM10 may represent the reason of decreased sLAG3 levels in the CSF of PD patients [40]. Hence, potential interactions between LAG3 and ADAM10 and ADAM17 metalloproteases in the case of PD should be also further investigated.

Regarding the role of LAG3 as a potential biomarker, clinicians should interpret data with caution for comorbidities that could possibly alter sLAG3 levels in the serum or CSF [40]. Of note, LAG3 protein could not bind to beta-amyloid PFF, beta-amyloid oligomer PFF or tau PFF, suggesting a relative specificity of LAG3 for aggregated α-synuclein [15]. However, given the fact that the two clinical studies discussed above excluded subjects with cancer, infections, tumors or other neurodegenerative disorders from the control group [7,40], future research is needed towards this direction.

Considering the crucial role of neuroinflammation in the neurodegenerative process of PD, targeting the immune responses represents a promising approach towards PD treatment [5]. The recently identified interaction between LAG3 and α-synuclein and its potential role in PD progression offers a novel therapeutic target. Several clinical trials have investigated LAG3-targeting molecules mainly in cancer immunotherapy [11]. In this context, IMP321, a novel recombinant LAG-3Ig fusion protein agonizing MHC II-mediated activation of dendritic cells, has been also tested in patients suffering from advanced renal cell carcinoma, and no significant adverse effects have been observed [76]. In regard to CNS, anti-LAG3 monoclonal antibodies are being currently investigated in the case of recurrent glioblastoma multiform, being able to cross the blood–brain barrier (Trial ID NCT02658981).

## 7. Conclusions

Although existing evidence on the role of LAG3 in PD pathogenesis is still scant, there is ongoing interest of its role in α-synuclein transmission and PD-related immune responses, as well as its biomarker potential. Further studies are needed in order to elucidate the molecular mechanisms underlying the implication of LAG3 in PD, considering its possible interaction with other molecules mainly affecting α-synuclein spreading pathology. Importantly, the fact that LAG3 protein was incapable to bind to beta-amyloid oligomers or tau suggests the relative specificity of LAG3 for PD compared to other neurodegenerative disorders, including AD. However, the identification of LAG3 as an α-synuclein PPFs-binding protein opens more avenues for the development of novel therapeutic strategies to slow the progression of PD. Given its important role in autoimmune activation in the brain, drugs or antibodies blocking specifically LAG3 are necessary to minimize the risk of side effects.
